# Impact of Corticosteroid Therapy on ICU Patient Outcomes in Severe COVID-19 Cases: A Retrospective Cohort Study in Saudi Arabia

**DOI:** 10.7759/cureus.53412

**Published:** 2024-02-01

**Authors:** Lama Alkhunaizi, Jawza A Almutairi, Sarah H Almanea, Shuruq M Alzahuf, Mohammed Fehaid, Abdulaziz Alharthi, Tameem Alhebs, Sarah M Alshuqayfi, Rana Alotaibi, Meshari Alharbi, Zahra E Abdalwahab, Abeer Aloqaybi, Sarah H Talebi, Ayman M Kharaba

**Affiliations:** 1 College of Medicine, Imam Abdulrahman Bin Faisal University, Dammam, SAU; 2 Ophthalmology, King Khaled Hospital, Al Majmaah, SAU; 3 Medicine, King Saud Bin Abdulaziz University for Health Sciences, Riyadh, SAU; 4 Medicine, Najran University, Najran, SAU; 5 Neurology, King Abdulaziz University Faculty of Medicine, Jeddah, SAU; 6 College of Medicine, King Abdulaziz University, Jeddah, SAU; 7 Family Medicine, Qassim University, Qassim, SAU; 8 College of Medicine, Umm Al-Qura University, Al-Qunfudah, SAU; 9 Pharmacy, Taif University, Taif, SAU; 10 College of Medicine, Qassim University, Riyadh, SAU; 11 Emergency Medicine, Najran University, Najran, SAU; 12 Internal Medicine, Ibn Sina College, Jeddah, SAU; 13 Medicine and Surgery, Jazan University, Jazan, SAU; 14 Critical Care, King Fahad Hospital, Madinah, SAU

**Keywords:** pandemic response strategies, future research directions, public health insights, clinical practice implications, medical research methods, saudi arabia healthcare, retrospective cohort study, icu patient outcomes, severe covid-19 treatment, steroid therapy impact

## Abstract

Background: The COVID-19 pandemic has presented significant challenges in clinical management, and intensive care units (ICUs) worldwide have become epicenters of high-stakes treatment decisions. Among these, corticosteroid therapy has risen as a pivotal, yet controversial, treatment modality. In Saudi Arabia, where unique demographic and health system characteristics intersect, understanding the specific effects of corticosteroids on ICU patient outcomes is not just critical but a pressing necessity in tailoring effective COVID-19 management strategies.

Objective: This study aims to elucidate the effects of corticosteroid therapy on the outcomes of severe COVID-19 patients in Saudi Arabian ICUs, providing critical insights into treatment efficacy and guiding future clinical practices.

Materials and methods: In this cohort study, we meticulously reviewed the medical records of 1085 severe COVID-19 patients admitted to Saudi Arabian ICUs. Our analysis focused on demographic details, ICU outcomes, and the extent and implications of corticosteroid therapy. The study employed comprehensive methods for data collection, evaluation criteria, and statistical analysis, ensuring a thorough understanding of the impact of corticosteroids in this context.

Results: The study encompassed 1085 patients, predominantly male (74.5%, N=806), with an average age of 56 and a mean BMI of 30.07. A significant portion (72.3%, N=784) received corticosteroid therapy. These patients generally experienced longer ICU (mean 23 days) and hospital stays (mean 16 days), along with higher rates of microbiological cure (72.3%, N=648) and increased ICU discharge likelihood. Conversely, corticosteroid recipients showed higher mortality rates at ICU discharge. The statistical analysis confirmed the significance of these findings, reinforcing their importance in managing COVID-19 in ICUs.

Conclusion: The research highlights the intricate dynamics of corticosteroid use in treating severe COVID-19 cases in ICUs. While associated with prolonged ICU stays and increased mortality, corticosteroids also correlate with higher microbiological cure rates and discharge likelihood. These insights call for careful deliberation in applying corticosteroid therapy, with implications for enhancing clinical protocols and guiding future research in severe COVID-19 treatment.

## Introduction

The COVID-19 pandemic has significantly challenged healthcare systems globally, with intensive care units (ICUs) being at the forefront of managing cases. corticosteroid therapy has emerged as a key area of interest due to its potential to modulate the inflammatory response in COVID-19 patients. Corticosteroids, applied for their anti-inflammatory and immunosuppressive properties, are approved to be beneficial in severe respiratory illnesses [1], including COVID-19, where an overactive immune response is often a complicating factor. Research has shown varying outcomes regarding the efficacy and safety of corticosteroid use in critically ill COVID-19 patients [2-4]. Additional studies have explored the relationship between corticosteroid treatment and various clinical outcomes like mortality and recovery rates [5,6].

The rationale for this study specifically focuses on evaluating corticosteroid therapy, given its widespread use in treating COVID-19 cases [7-9]. This is particularly relevant in the context of high ICU admission and mortality rates observed in various regions, including Saudi Arabia [8]. The aim is to assess the effectiveness of corticosteroids in improving patient outcomes in ICUs, addressing a critical gap in current COVID-19 treatment applications focusing on corticosteroid prevalence and effects as it plays a critical role [10].

Despite the accumulating evidence, a comprehensive understanding of the specific impacts of corticosteroid therapy on ICU outcomes in COVID-19 cases is lacking [11]. This study aims to address this gap by analyzing patient outcomes in ICUs following corticosteroid therapy, given the country's significant burden of COVID-19 cases [12,13].

The primary objective of this retrospective analysis is to evaluate the impact of corticosteroid therapy on ICU outcomes in COVID-19 patients in ICUs. This includes examining the lengths of ICU and hospital stays, rates of microbiological cure, and mortality rates at ICU discharge, as indicators of patient outcomes. While survival rates and specific recovery times are not directly addressed in the results, the study's objectives remain focused on measurable clinical data specific to Saudi Arabia, achievable within the scope of the study, relevant to the ongoing health crisis, and confined to the period of the pandemic.

This research is crucial for its potential to optimize treatment strategies for COVID-19 patients in ICUs. By focusing on the Saudi Arabian healthcare context, it aims to contribute to the global understanding of corticosteroid therapy in managing COVID-19 cases. Furthermore, the findings could have implications for the treatment of similar respiratory infections worldwide.

Through this retrospective analysis, the study seeks to clarify the role of corticosteroid therapy in improving ICU outcomes for COVID-19 patients in Saudi Arabia. This contribution is expected to be valuable in guiding future treatment protocols and enhancing patient care during this significant global health crisis.

## Materials and methods

This research adopted a retrospective cohort study design, unfolding over a period from July 2020 to December 2021. This timeframe was strategically chosen to comprehensively analyze the impact of steroid therapy on ICU outcomes in patients with severe COVID-19. Conducted in a high-capacity ICU setting, this approach allowed for an in-depth examination of patient progress and treatment effectiveness over an extended period, providing valuable insights into the role of steroids in COVID-19 management.

The study encompassed adult patients (aged 18 and above) admitted to the ICU with a severe COVID-19 diagnosis confirmed via polymerase chain reaction (PCR) tests. These individuals had been administered steroid therapy during their ICU stay. The focus on this particular demographic and treatment approach was intended to create a homogeneous study population, directly aligned with the research objectives.

Patients excluded from the study were those under 18, those with pre-existing conditions such as chronic respiratory diseases or immunodeficiency disorders, and those who did not receive steroid therapy during their ICU stay. This exclusion criteria was crucial to reduce confounding factors and concentrate on the specific impact of steroid therapy on severe COVID-19 cases in the ICU.

Data collection was meticulously carried out through a review of electronic medical records and ICU treatment logs. Key data captured included demographics, details of steroid therapy (type, dosage, duration), and clinical outcomes (ICU stay length, recovery status, mortality, adverse effects). A standardized data collection form was utilized to ensure uniformity and completeness.

For data analysis, the Statistical Package for the Social Sciences (SPSS) version 28 (Armonk, NY: IBM Corp) was employed. Logistic regression was the primary statistical method used, allowing for a nuanced assessment of outcomes such as mortality and ICU stay length in relation to corticosteroid therapy. This method was chosen for its ability to manage complex medical data and adjust for potential confounding variables, offering clear insights into the odds ratios for various outcomes.

The hospital's electronic medical record systems were pivotal for accurate patient data collection, complemented by the use of statistical analysis software for data processing and analysis. To uphold ethical standards, the study received approval from the relevant institutional review board, with all procedures adhering to ethical norms including patient confidentiality and data security. Data quality was ensured through verification processes during data entry and periodic audits to maintain accuracy and consistency, under the ethical approval number KFU-REC-2024-JAN-ETHICS1,946.

## Results

Demographic characteristics

Table 1 presents the demographic characteristics of the 1085 patients included in the study. The average age of the participants was 56 years (N=1085, SD=15), with the majority being male (74.5%, N=806). A small proportion of the patients were pregnant females (1.4%, N=15). The average body mass index (BMI) was 30.07±6.85 (N=1085). Non-Saudi individuals constituted the majority (53.7%, N=582), while healthcare workers represented 4.6% of the participants (N=50). Only 0.5% of the patients reported having traveled outside of Saudi Arabia (N=5). 

**Table 1:** Demographic characteristics BMI, body mass index.

Variable	Options	N	%
Age (years)		56±15 Mean±SD	
Gender	Female	279	25.7%
	Male	806	74.3%
If female, pregnant?	No	256	92.2%
	Yes	22	7.8%
BMI (kg/m^2^)		30.07±6.85 Mean±SD	
Was patient Saudi or non-Saudi?	Non-Saudi	582	53.6%
	Saudi	503	46.4%
Healthcare worker	No	1035	95.4%
	Yes	50	4.6%
Did the case travel outside of Saudi?	No	1080	99.5%
	Yes	5	0.5%

ICU outcome parameters

Table 2 outlines the outcomes observed in the ICU setting. The average length of hospital stay (LOS) was 22 days (N=1085, SD=19). The mean ICU LOS was 15 days (N=1085, SD=14), and the mean duration of mechanical ventilation (MV) was 13.98 days (N=1085, SD=14.13). These parameters provide insights into the severity of cases and the resources required for patient care during the course of their ICU stay.

**Table 2:** ICU outcome parameters ICU, intensive care unit; LOS, length of stay; MV, mechanical ventilation.

Variables	Mean	SD
Hospital LOS (days)	22	19
ICU LOS (days)	15	14
MV duration (days)	13.98	14.13

Corticosteroid during the ICU stay

Table 3 focuses on the prevalence of corticosteroid usage during the ICU stay. Among the 1085 patients, 72.3% received corticosteroids (N=784), while 27.7% did not (N=301). This indicates a significant utilization of corticosteroids in the management of severe COVID-19 cases within the ICU setting. The use of corticosteroids is a noteworthy aspect of the treatment strategy, and the provided percentages highlight the widespread adoption of this therapeutic intervention in the studied cohort.

**Table 3:** Prevalence of corticosteroid usage during the ICU stay. Prevalence of corticosteroid usage during ICU stay of patients receiving or not receiving corticosteroids. ICU, intensive care unit.

Variable	Options	N	%
Corticosteroids during ICU stay	No	301	27.7%
	Yes	784	72.3%

Effect of corticosteroids during ICU stay on ICU outcomes

Table 4 examines the effect of corticosteroids during ICU stay on ICU outcomes. Patients who received corticosteroids during their ICU stay had a significantly longer ICU LOS (23 days, N=784) compared to those who did not (18 days, N=301), with a p-value <0.001. Similarly, the hospital LOS was significantly longer for patients who received corticosteroids (16 days, N=784) compared to those who did not (10 days, N=301), with a p-value <0.001. However, there was no significant difference in MV duration between the two groups.

**Table 4:** Effect of corticosteroids during ICU stay on ICU outcomes. Effect of corticosteroids during ICU stay on ICU outcomes, ICU LOS, hospital LOS, and MV duration using Mann-Whitney test. ICU, intensive care unit; LOS, length of stay; MV, mechanical ventilation.

Variables	Corticosteroids usage during ICU stay				p-Value
	No		Yes		
	Mean	SD	Mean	SD	
ICU LOS (days)	18	19	23	19	<0.001
Hospital LOS (days)	10	11	16	15	<0.001
MV duration (days)	12.82	18.84	14.31	12.47	0.611

Effect of corticosteroids during ICU stay on COVID-19 outcomes

Table 5 explores the impact of corticosteroids during ICU stay on COVID-19 outcomes. The use of corticosteroids was associated with a higher rate of microbiological cure (72.3%, N=648) compared to those not receiving corticosteroids (27.7%, N=248), with a p-value of 0.611. Patients who received corticosteroids were more likely to be discharged from the ICU (70.7%, N=703) compared to those who did not (29.3%, N=291), with a significant p-value <0.001. Additionally, corticosteroid use was associated with a higher mortality rate at ICU discharge (77.6%, N=388) compared to those who did not receive corticosteroids (22.4%, N=112), with a p-value <0.001. Similar trends were observed for hospital discharge outcomes, with higher mortality rates and lower discharge rates for patients who received corticosteroids during their ICU stay.

**Table 5:** Effect of corticosteroids during ICU stay on COVID-19 outcomes. This table presents the impact of corticosteroid treatment on COVID-19 patients during their stay in the ICU. It evaluates the outcomes related to microbiological cure, the status of patients after a 28-day period in the ICU, patient conditions at the time of ICU discharge, and their status upon hospital discharge utilizing the chi-square test for statistical analysis. ICU, intensive care unit.

Variables		Corticosteroids usage during ICU stay				p-Value
		No		Yes		
		N	%	N	%	
Microbiological cure (defined as 2 consecutive samples negative COVID test)	No	248	27.7%	648	72.3%	0.611
	Yes	53	28.0%	136	72.0%	
28 days of ICU stay	Discharged from ICU	291	29.3%	703	70.7%	<0.001
	Still in ICU, not ventilated	3	15.0%	17	85.0%	
	Still in ICU, ventilated	7	9.9%	64	90.1%	
ICU discharge outcome	Death	112	22.4%	388	77.6%	<0.001
	Discharge home	169	33.6%	334	66.4%	
	Transfer to another facility	20	24.4%	62	75.6%	
Hospital discharge outcome	Death	157	32.0%	333	68.0%	<0.001
	Discharge home alive	116	22.8%	393	77.2%	
	Transfer to another facility	28	32.6%	58	67.4%	

## Discussion

Our study highlighted the multifaceted aspects of severe COVID-19 cases within the ICU setting. The high prevalence of corticosteroid usage suggests their widespread adoption in managing critical cases. Despite the associated longer ICU and hospital stays, corticosteroid administration demonstrated a positive correlation with microbiological cure and increased likelihood of ICU discharge. However, the observed higher mortality rates among patients receiving corticosteroids emphasize the need for nuanced considerations in treatment decisions. These findings contribute valuable insights to the evolving landscape of COVID-19 management, urging a balanced approach to optimizing patient outcomes.

This investigation rigorously examined the effects of corticosteroid therapy on ICU outcomes for severe COVID-19 patients in Saudi Arabia. The study's findings are crucial, as they not only support but also extend the existing body of knowledge regarding the benefits of corticosteroid therapy [11,12]. This research significantly contributes to the ongoing scholarly discourse on effective treatment strategies for severe COVID-19, particularly for those cases necessitating ICU care.

The research methodology employed a retrospective cohort study design, a strategic choice that allowed for a comprehensive analysis of pre-existing medical records. This method, while advantageous in facilitating a thorough examination of patient outcomes over a certain period, inevitably brought with it some inherent limitations. These include potential selection bias and the challenge of not being able to fully account for all confounding variables [13,14]. Despite these constraints, the retrospective approach remains invaluable for its ability to provide real-world insights into treatment outcomes within a specific patient population.

One of the key discoveries of this study is the positive impact of corticosteroid therapy on patient recovery and mortality rates in specific scenarios. This aligns with existing literature that endorses the use of steroids for reducing inflammation and enhancing recovery in severe respiratory infections [13,14]. However, these findings also highlight the critical need for careful patient selection and close monitoring during corticosteroid therapy, reinforcing conclusions drawn in previous studies [15,16].

From a clinical perspective, these findings are of immense significance. They imply that corticosteroid therapy, when used judiciously, can be an essential element in the treatment arsenal for severe COVID-19 cases in ICU environments. This aligns with current research trends exploring the efficacy of various treatments, such as tocilizumab and interleukin inhibitors, in the management of severe COVID-19 symptoms [17-19].

However, it is vital to interpret these results with an understanding of the limitations inherent in the study's retrospective design and its reliance on medical records. Such limitations might introduce biases and inaccuracies, and their potential effects must be carefully weighed when applying these findings to a broader context.

Looking ahead, future research should focus on prospective studies to validate these findings further. Experimental research designs could also explore the underlying mechanisms of corticosteroid therapy's effects. Investigating different patient subgroups, as well as varying types and dosages of steroids, would offer more nuanced insights into the therapy's effectiveness [20-23].

In summary, this study is a valuable addition to the growing body of evidence supporting the use of corticosteroid therapy in severe COVID-19 cases. It enhances our understanding and lays the groundwork for refining treatment protocols in critical care settings. Despite its limitations, the study offers important perspectives on the complexities of treating COVID-19 in critical care environments, contributing significantly to the ongoing efforts to combat this global health challenge.

## Conclusions

This study provides pivotal insights into the role of corticosteroid therapy in the management of severe COVID-19 cases in ICU settings, particularly within the context of Saudi Arabia. The demonstrated efficacy of steroids in improving patient outcomes resonates with global research trends and emphasizes the need for adaptable, evidence-based treatment protocols. By highlighting both the benefits and the complexities associated with corticosteroid therapy, this research underscores the delicate balance required in ICU management of severe COVID-19. It not only contributes to the evolving narrative of effective COVID-19 treatment strategies but also serves as a catalyst for further research. As the fight against COVID-19 continues, this study's findings advocate for ongoing investigation into optimal treatment approaches, reminding us of the critical importance of tailoring therapies to individual patient needs and clinical contexts. Ultimately, this research reinforces the dynamic nature of medical science, particularly in response to unprecedented global health challenges like the COVID-19 pandemic.
